# Developing Future Deep-Space Telecommunication Architectures: A Historical Look at the Benefits of Analog Research on the Development of Solar System Internetworking for Future Human Spaceflight

**DOI:** 10.1089/ast.2018.1915

**Published:** 2019-03-06

**Authors:** Marc A. Seibert, Darlene S.S. Lim, Michael J. Miller, Delia Santiago-Materese, Michael T. Downs

**Affiliations:** ^1^ ASRC Federal, NASA Headquarters, Washington, District of Columbia.; ^2^ NASA Ames Research Center, Moffett Field, California.; ^3^ Bay Area Environmental Research Institute, Moffett Field, California.; ^4^ NASA, Kennedy Space Center, KSC, Florida.

**Keywords:** BASALT, Human exploration, Extravehicular activity, EVA, Deep-space, Telecommunication, Space communication, Latency, EVA informatics, Astrobiology, Astrogeology

## Abstract

Exploration analog field tests, missions, and deployments enable the integration and validation of new and experimental concepts and/or technologies through strategic experimental design. The results of these operations often create new capabilities for exploration and increase confidence in, and credibility of, emerging technologies, usually at very low cost and risk to the test subjects involved. While these experiments resemble missions 10–30 years into the future, insights obtained are often of immediate value. Knowledge gained in the field translates into strategic planning data to assist long-range exploration planners, and planners influence the experimental design of field deployments, creating a synergistic relationship. The Biologic Analog Science Associated with Lava Terrains (BASALT) communication architecture is a high-fidelity analog program that emulates conditions impacting future explorers on the martian surface. This article provides (1) a brief historical review of past analog operations that deliberately used elements of a flight-like telecommunication infrastructure to add fidelity to the test, (2) samples of the accomplishments made through analog operations, and (3) potentially significant deep-space telecommunication insights gained from the BASALT program in support of future extravehicular activity exploration of Mars. This article is paired with and complements Miller *et al.* in this issue which focuses on the telecommunication infrastructure utilized by the BASALT team during the field deployment.

## 1. Introduction

Analog field tests of human and robotic planetary exploration missions predate the Mercury missions (Phinney, 2015) and they have been essential to efficient and safe mission planning (Hettrich *et al.*, 2015). Early field tests were primarily for testing prototype flight hardware, tools, suits, procedures, and people. Modern analogs perform the same tests as their predecessors, but also include rigorous testing and validation of many new approaches for missions that will occur decades into the future, through integrated and carefully designed experiments of varying size and scope. NASA published several documents about active analogs up to 2011 (Craig *et al.*, 2011; Ross *et al.*, 2013).

Analogs help to address critical questions facing policy-makers and mission planners who would otherwise be forced to make decisions with little to no available data. NASA's Space Communications and Navigation (SCaN) Program has been one of the number of beneficiaries of the results from analog field deployments. Analogs not only assist these decision makers with tangible results that have multidecade impacts but also often yield unprecedented, and sometimes unexpected, insights of immediate value to long-range planners and cost analysts. These individuals benefit from this timely information because they often need decades to fiscally prepare for, procure, deploy, test, and commission in-space infrastructure for future spaceflight missions.

There are many organizations conducting space exploration analog research, from a variety of countries. Many of these analogs are multifaceted, focusing on a multitude of components related to human exploration activities and time lining. Analogs are typically low-cost, flexible, and highly efficient operations that emulate actual space missions, because many systems and subsystems at various stages of development can be validated at once. They can enable teams to integrate with one another, gain a wider exposure and peer review of their activities, and obtain feedback from unbiased users on the strengths and weaknesses of their part of the operation.

Our article examines known analogs that have been active over the last decade and that have deliberately included protoflight telecommunication infrastructures. This is in direct contrast to analogs that simply used communications infrastructure to conduct a field test. The analogs we have focused on are a small fraction of the vast number of analog missions funded by both NASA and by other international agencies. Our goal is to provide a historical foundation that serves to identify existing knowledge gaps in the space communications community and to provide a reference point for future analog activities.

Many questions remain in the space communications community regarding future deep-space data transmission requirements, and as spaceflight to Mars is added to the portfolio of missions, even more questions materialize. Evaluating new options for future deep-space telecommunications and space Internetworking by using analog missions is both challenging and highly rewarding in terms of filling knowledge gaps and identifying process and hardware requirements. Often the success of the analog mission team is highly dependent on a reliable telecommunication infrastructure because other teams create and rely on the delivery of valuable data “payload” that must be carefully carried by any experimental telecom architecture. Like spaceflight missions, when the communications network at an analog is “down,” everyone's ability to work is likely compromised. Test timelines may halt, and minutes, hours, or days of testing can be lost completely.

Despite this risk, the telecom team must also learn from the experience, especially because of how far ahead in-space infrastructure decisions must be made before missions. Space Internetworking and telecommunication system developers need environments to test the efficacy of their systems in real environments in order to understands their capabilities and weaknesses at analogs they must serve as part “service provider” and part “protoflight telecom capability tester.” Risky protoflight telecom infrastructure must have a backup ready or engage a fallback mode if the experimental system proves unreliable.

Fortunately, over the last decade, a handful of analog teams have tolerated various levels of risk and experimentation by their telecommunication subteams, so they too could benefit from the opportunity analog testing provides the participants. The following is an overview of these innovative analog test environments.

## 2. The Early Years of Terrestrial System Development for Spaceflight

The first known fieldwork that resembled a human spaceflight analog predates the first Mercury missions by a few years (Phinney, 2015). The processes that led up to the first spaceflight-relevant analogs are best described by Phinney,

At first, the initial training for the astronauts, whose task was primarily to fly spacecraft, was thought to be similar to that of test pilots who needed to learn how to use the controls and instruments required to fly their craft. As more thought was put into the nature of the training required for flying spacecraft a training program was designed to include some of the scientific needs that might be necessary in this new endeavor.

….

The first of these science training programs started in May 1959 and included mechanics and aerodynamics, space physics, principles of guidance and control, navigation in space, elements of communication, and basic physiology. Each astronaut also spent time at a planetarium studying star recognition and celestial navigation.

…

The USGS had programs of lunar mapping and crater studies well under way by the early 1960s. … By 1965 the USGS had formally proposed most of the types of equipment considered essential for samp1e selection, collection, and documentation on the lunar surface, including a scoop, extension handle, core tubes, hammer, scriber/brush, hand lens, sample bags, and a tool and sample carrier. (MSC had already started development of most of these tools through a contract with Martin Marietta in 1964.)

…

The USGS also had developed radio and television (TV) communication systems between field crews and a central control facility. In addition, the USGS, along with personnel at [the NASA Manned Spaceflight Center/NASA Johnson today], conducted many geological tests in pressure suits to determine the extent to which mobility and exhaustion would affect the ability to conduct geologic activities on the lunar surface.

…

Furthermore, they began developing equipment and procedures for a more extended exploration program to follow Apollo. It was during the growth of these organizations that the detailed plans for further training of the astronauts were developed.

**FIG. 1.**
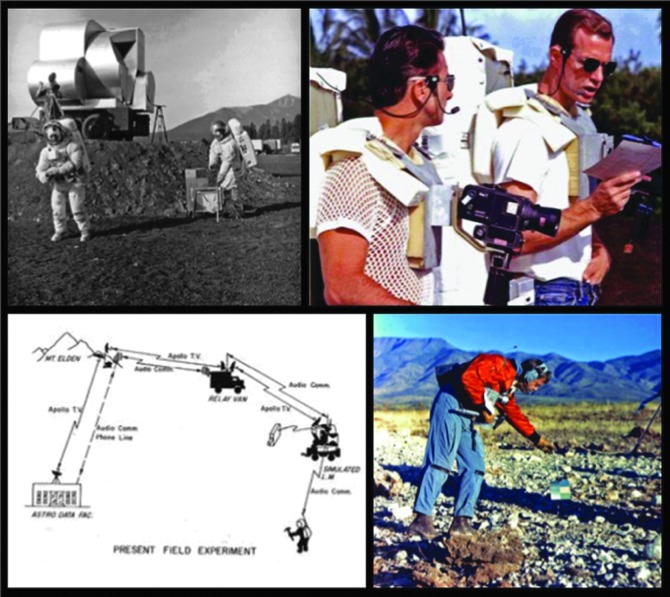
Top row and lower right: Apollo crewmembers performing what we today call analog missions, for testing procedures, concepts, equipment, and training crews. Lower left: Apollo-era telecommunication link diagram closely resembles modern field test telecomm link diagrams (Schaber, 2005).

Field testing conducted by the Apollo program and crews (Fig. 1) focused mainly on training systems, tools, procedures, and people that were already planned for an upcoming flight and were not focused on evaluating entirely new future concepts.

Finney also provides insight into the protoflight telecommunication infrastructure used at the first Apollo field tests, and the field test process in general,

During the actual field exercises the crews would be in voice communication with the Capcoms in makeshift backrooms that were set up in the field and all conversations were taped.

…

Upon completion of each exercise a walkthrough debriefing was carried out for all participants in the exercises to determine how well the crews did on the exercise, how well the tools and other equipment performed, and how well the procedures worked.

…

Upon return to Houston, or elsewhere, the collected samples were laid out, the tapes were transcribed, and the photos developed in preparation for a detailed critique with the astronauts. The photos and transcripts were correlated with the samples and further related to the geologic features that were studied. This would include notes on the evaluation of photography, adequacy of samples, and completeness of descriptive information.

…

The field exercises also provided an opportunity to fine-tune any problems with the hand tools, cameras, and other equipment.

…

During the field exercises there were personnel to provide and maintain the hand tools and cameras. Problems with these instruments were always documented and either corrected on the spot or slated for further study upon return.

**FIG. 2.**
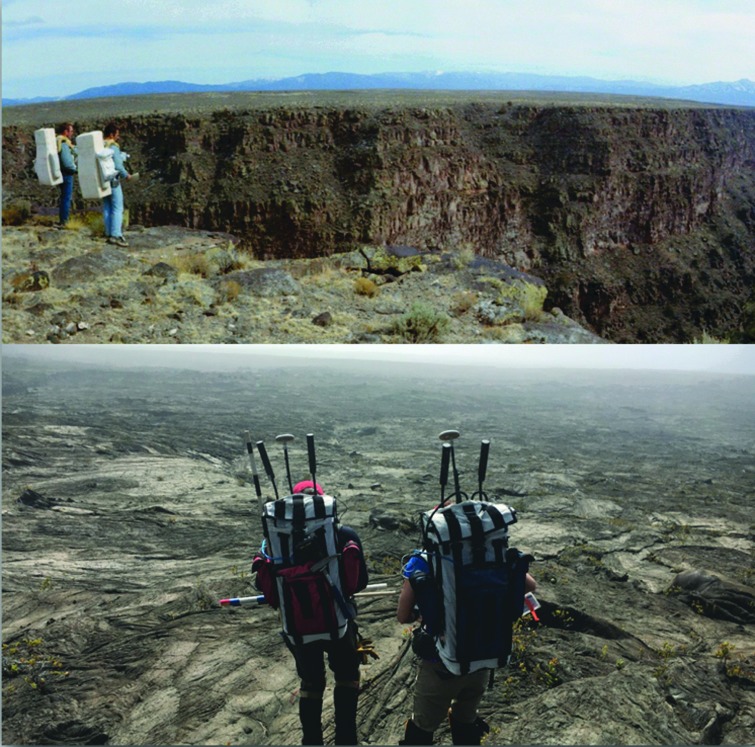
Then and now. Top: Apollo 15 CDR David Scott (left) and James Irwin (right) during practice geologic EVA training at the Rio Grande Gorge, Taos, NM, March 1971 (NASA Photograph AS15-S71-23773). Bottom: Two BASALT geologists during Hawaii field deployment (photo credit: Miller/Beaton/Elphic). BASALT, Biologic Analog Science Associated with Lava Terrains; EVA, extravehicular activity.

The Apollo program field testing was designed for a different purpose than most analogs today. The testing focused on training the selected crews for a specific scheduled upcoming mission; however, these analogs possessed some similarities to their modern counterparts, and at times even “looked” similar (Fig. 2). The scientists and engineers learned ways to improve their processes, but they were limited in how far afield of mission training they could skew to explore new ideas and concepts for other mission years or decades ahead.

In contrast to the field testing of the past, modern analog deployments such as those conducted by the Biologic Analog Science Associated with Lava Terrains (BASALT) research program (see Lim *et al.*, 2019, for overview) and many others (Craig *et al.*, 2011) focus on key elements of future human/robotic exploration missions that may occur in the 10- to 30-year time frame. At the time of publication, the BASALT program is currently conducting the highest fidelity simulations of future scientific extravehicular activity (EVA) operations on the Moon and Mars.^*^

## 3. Evolution of Field Test Support Infrastructure into Architectures of Protoflight Systems

### 3.1. NASA robotic analogs between 1985 and 1997

NASA conducted many groundbreaking analog field test activities between 1985 and 1997. The field work focused primarily on automation, robotics, telerobotic operations, and the advancement of machine learning and artificial intelligence (Holcomb and Montemerlo, 1987). Some tests included some human interaction with the robots simulating aspects of human/robotic exploration (Weisbin and Montemerlo, 1992). In 1992, a NASA/Carnegie-Mellon University team deployed a pair of robots named Virgil and Dante to Mount Erebus in Antarctica (Wettergreen *et al.*, 1993). The rovers were teleoperated by a team locally over a 2-km-long fiber optic cable (wireless data links at that time were cost-prohibitive), and the control center was linked with a control team at Goddard Space Flight Center (GSFC) via Tracking and Data Relay Satellite (TDRS) link. The team at GSFC was able to watch the rover's cameras and they could teleoperate the rovers when they remotely controlled the robot control workstations at the site (Wettergreen *et al.*, 1992). Robotic development and operation activities during this time frame helped mature the knowledge, systems, and operation capabilities needed to enable the first robotic rovers to explore the surface of Mars, starting with the Pathfinder rover that landed on Mars in December 1996 (Lindemann *et al.*, 2005).

### 3.2. Key international participants

Exploration analog research is conducted by numerous organizations within the United States, including a variety of universities and various NASA programs. NASA-funded analog work has been and continues to be supported by many programs within the agency. The Canadian government funds exploration analog activities active today and has for decades. The funding and objectives have largely been managed by personnel at the Canadian Space Agency (CSA) (Osinski *et al.*, 2006) and CSA maintains an accounting of over 50 different high-quality analog sites for scientific sampling or teams to utilize for analog field testing (Cloutis *et al.*, 2015). Russia and countries within the European Space Agency also conduct analog space missions (Groemer *et al.*, 2014).

### 3.3. Extreme human isolation with low- to modest-fidelity analog telecommunication systems

Expeditions to the Antarctica have long been considered analogous to space exploration in many ways (Hoffman, 2002). Scientists in NASA's Human Research Program (HRP) have provided compelling evidence that the isolation experienced by teams working in the Antarctica provide a very high-fidelity analog for some aspects of deep-space exploration—in particular, insights into the psychosocial effects that long-term isolation has on small work teams (Slack *et al.*, 2016). The first telecommunication link to McMurdo Station near the South Pole, Antarctica, was a single, analog video link with bidirectional audio tested by NASA in November 1992 (Leon, 1995). The McMurdo link has been upgraded regularly as new satellite options overhead have emerged. Today, McMurdo Station enjoys on average 3.5 h of satellite communications (“satcom”) connectivity daily using a bent-pipe satellite link through the Defense Satellite Communications System-3 B7 satellite to the program hub in Denver, CO. Aggregate/shared bandwidth rates for all explorers/users of the Internet connection at McMurdo average 30 Mbps down, 10 Mbps up—when the satellite is in view (National Science Foundation—US Antarctic Program [NSF-USAP], 2018). This aggregate link is distributed across a local wired and wireless network at the station.

The Hawaii Space Exploration and Analog Simulation (HI-SEAS) Project habitat (Binsted *et al.*, 2016) is geographically closer to civilization, but the test subjects experience isolation similar to those on McMurdo missions. HI-SEAS focuses on the long-duration habitation phase of exploration and its effects on the human explorers. HI-SEAS is helping to develop a better understanding of the biological, psychological, and social aspects impacting small teams of crews isolated from the rest of society for very long periods of time (8–12 months per mission). The test subjects can only communicate with the rest of humanity over 20-min delayed two-way communication links. This constitutes the worst possible scenario when Earth and Mars are near conjunction (although HI-SEAS does not simulate the 2–3 weeks of total communication outage that occurs regularly between Earth and Mars during actual conjunction). The missions are carefully designed and controlled human studies framed in a Mars mission context. Test subjects are volunteers from the general public (internationally) who are down-selected to be crewmembers and establish a mission role/title determined by their experience and interests, and crews vote to select their commander before the mission.

HI-SEAS has completed five missions as of the date of printing of this article, ranging in duration from 3 to 12 months, and each mission has gradually increased the protoflight fidelity of the telecommunication infrastructure supporting the crew. The habitat is situated in a remote location halfway up the side of Mauna Loa volcano (8000′ elevation), where the weather can be unpredictable, adding to crew stress on occasion. The habitat today has two broadband telecommunication links: high- and low-rate links serving as prime and backup, which is analogous to a future Mars relay architecture to a surface habitat. The crew also has a cellular telephone in the habitat restricted to use only in emergencies, as required by the Institutional Review Board (IRB) that approves and monitors the safety of each mission. However, even this cellular phone has a Mars analog. In an emergency situation on Mars where other communication paths went down to the habitat (which has happened on occasion during HI-SEAS), the crew would likely power on a high-powered communication link direct to Earth, if visible, which is similar to a HI-SEAS crew either texting or placing a call on the emergency cell phone, to request help. The HI-SEAS crew also exchanges files in *a delayed manner* with the International Mission Support team, all e-mail is delayed (20 min, each way), and the crew can only browse a short list of approved Internet domains and websites. The white-listed website access simulates a volume of web pages that will 1 day be cached to one or more servers on Mars, so the pages can be browsed locally and successfully by the exploring crew. HI-SEAS mission 3 had the most instrumented network to date, and many insights were captured from the measurement of data products during the mission. Like the BASALT program, the insights from the field were fed directly into a NASA SCaN Program deep-space capacity study currently underway (Seibert *et al.*, 2017a, 2017b).

### 3.4. First EVA “informatics” testing with modest-fidelity local surface wireless networks

Informatics is the science of information systems. In the case of space suits, informatics means high-power minicomputers were placed on simulated suits to evaluate how information systems can enhance exploration. The first known EVA-worn informatics systems to be field tested in a space exploration context (one that included modern computer technology with experimental software) emerged in the primarily NASA-funded Haughton-Mars Project (HMP) in 2001 (Boucher *et al.*, 2002). The HMP site, located on Devon Island in the High Arctic of Canada started hosting researchers in 1997 (Lee, 2002), celebrated its 20th field deployment in 2015 (Lee and Crawford, 2017). The PolyLAB at Simon Fraser University is known for some of its early work in telemedicine set in an exploration context (Braham *et al.*, 2001), and is believed to be the first organization to experiment with a system to impose a real Mars-delayed telecommunication link for voice communications made over links to an active analog site (Boucher *et al.*, 2002).

### 3.5. Advanced human/robotic systems testing with modest to high telecommunication fidelity

NASA began testing advanced EVA suit mobility in the field in May 1998 (Kosmo and Ross, 1999). The pressurized suit was run through a variety of tests in multiple desert locations simulating lunar and martian terrains outside of Flagstaff, AZ, with support from the US Geological Survey staff based in Flagstaff. In February the following year (February 22–27, 1999), the team partnered with a team of robotic engineers and performed the first integrated human/robotic exploration simulation at Silver Lake, in the Mojave Desert (Trevino *et al.*, 2000). This human/robotic exploration research team continued to grow and was named the NASA Desert Research and Technology Studies (D-RATS) team in 2002. This analog activity focused on integration of the exploration systems.

The first three D-RATS missions conducted operations with a short-range analog/FM voice intercom/radio system (1998–2000), a wireless suit camera (2000), and the EVA Robotic Assistant (ERA) robot was operated over wireless IEEE 802.11 (IEEE 802.11 Working Group, 1997) standard radios (1999–2000). The team operated independently and had no connectivity beyond the test site during missions 1–3, except for marginal cellular telephone coverage in some locations.

The fourth D-RATS mission (2002) was the first D-RATS test to include a broadband satellite link to the site (Ross *et al.*, 2004). This link was facilitated by the team driving a trailered, 3 m parabolic reflector antenna (and accompanying modems, up/downconverters, and other electronics) to the field, procuring transponder time from a satellite broker, and aligning the system with an assigned commercial Ku-band satellite overhead in geostationary orbit. Once the antenna was set up at the site, a 12 m antenna at the NASA Glenn Research Center also locked on to the same Ku-band commercial satellite, and the satellite operated in bent-pipe mode, providing 5 Mbps of network bandwidth (each way) between the test site and the ground station at NASA Glenn. Bent-pipe mode is when a telecommunication satellite immediately retransmits a signal it receives to another location with little change to or processing of the signal or information. The D-RATS field data network/circuit at Glenn was tied into NASA's Research and Engineering Network (NREN), which provided the field team access to various internal NASA servers, facilities, and the public Internet (NASA Research and Engineering Network [NREN], 2018). The satellite trailer in the field included an early-generation commercial network firewall, and the firewall successfully alerted and shielded the field team from multiple inbound network attacks each year. This same link was deployed for many years that followed and was also requested by other analog field teams such as the Mars Society for use at the Mars Desert Research Station (MDRS) test site in 2004. This link enabled the field team to exchange video, voice, and telemetry data back through NASA's internal networks to the Exploration Planning and Operations Center (ExPOC) for the first time. The ExPOC was a facility in the JSC Mission Control Center building established and utilized in the 2001–2008 timeframe, designed to test future mission operations with deep-space crews. Concurrently, the HMP team and the MDRS team also had experimental satellite communication systems at their test sites but were operating at lower data rates.

By the end of D-RATS mission 5 (2003), the team became more dependent on wireless communication systems. Like the other analog teams working in the field, they recognized that the challenges they experienced maintaining links and moving data wirelessly would also affect future explorers. At the time, NASA's Constellation program was focused on sending humans back to the Moon by 2020 (US President Bush, 2004), and so the D-RATS and other telecommunication subteams on analog projects decided to embrace the reality of communication challenges in lunar-relevant terrains. The telecommunication team evolved from *limiting operations to accommodate the challenges to the communication infrastructure* to *accepting the challenge of the terrain on communications* and working to help identify and test new infrastructures that more closely resembled what NASA would deploy on a planet surface. After about 5 years in the field, the D-RATS telecommunication team had gradually evolved the field architecture to a point where the project architecture had reached a point that was useful to SCaN office planning.

At the same time, the IEEE 802.11 wireless networking family, which was used in the field to connect devices such as on-suit/rover computers, cameras, and other telemetry devices short range, and the “WiMAX” IEEE 802.16 standard (IEEE 802.16 Working Group, 2005), which enabled links to 10s of kilometers, both evolved significantly. Many highly competitive and diverse products entered the commercial market, and the products provided steadily increasing data rates at growing link ranges. Some were even designed and proven to operate at very long ranges in the field (>20 km). At a time when cellular towers struggled to move data at rates up to 1 Mbps to mobile users in urban areas, the D-RATS telecommunication team was moving data at aggregate rates up to 10 Mbps at link ranges up to 22 km in support of the mobile field test operations (Hörz *et al.*, 2013). The longest surface-to-surface link the D-RATS team ever tested was put in place in the fall of 2004 between the summit of Mt. Elden in Flagstaff (where the team secured access to a government telecommunication interface) and the Joseph City test site. The test was in preparation for the fall 2005 field test, which tentatively was planned for Joseph City, but later occurred at the Cinder Lake field adjacent to Mt Elden. The equipment used antennas smaller than 1 m in diameter and were aligned manually by the team and successfully closed a multimegabit data link over a 120 km (75 miles) range.^†^

## 3.6. Project VELOCIRAPTOR: remote geologic exploration, high-fidelity telecommunications, and real-time Earth computing support of the explorers

The D-RATS telecommunication team also supported a one-time exploration analog field experiment in 2004, relevant here, code-named “VELOCIRAPTOR” (Beck *et al.*, 2005; Vincent and Beck, 2005). The Velociraptor project included several key elements: a field team of geologists with a handheld hyperspectral instrument and a data link back to NASA networks, two large computing clusters called “computing grids” interlinked but located at two NASA centers, an order for a scheduled EO-1 imaging pass, and the EO-1 support team standing by to expedite the satellite imaging for the Velociraptor team. Velociraptor's goal was to demonstrate how high-power computing grids (“cloud computing” today) on Earth^‡^ could provide useful support to Mars explorers by processing and returning data to crews on Mars before they returned home. The Velociraptor team demonstrated that explorers on the planet surface could help ground truth hyperspectral imagery taken by an orbiter overhead, and the computing grids could merge the orbital and ground-based imagery to generate and provide to the crew more accurate surface maps of specific geologic features found around the landing site. Some of the maps produced were specialized “binary” maps (two-color black and white maps) where the white regions on the maps revealed high-likelihood locations where specific geologic features could be found (kaolinite was one of several types mapped, *e.g*.). The Velociraptor scientists believe that maps of this type could help maximize the use of EVA time line, by empowering the explorers and their science support teams (SSTs) on Earth to very efficiently target exploration traverse planning based on the hypotheses of the interest each day or EVA.

## 4. Exploration Analogs and Deep-Space Infrastructure Planners

NASA's SCaN office (National Aeronautics and Space Administration, 2018a) is responsible for providing communication and navigation services near Earth or in Deep space^§^ to all NASA missions, and many non-NASA missions via support requests. SCaN uses three networks to provide coverage both near Earth and in deep space. The boundary between near-Earth and deep space is defined by the International Telecommunication Union (ITU) (International Telecommunication Union Recommendation [ITU-R], 2014). The three networks in use today are a combination of ground and space-based facilities and infrastructure provided by the Deep Space Network (DSN), Near Earth Network (NEN), and the Space Network (SN). Over time, the analog teams and the SCaN planners recognized a potential for mutual benefit from collaboration in planning, execution, and sharing of results. This relationship became somewhat synergistic in nature—both entities provided and received information resulting from the collaboration.

**FIG. 3.**
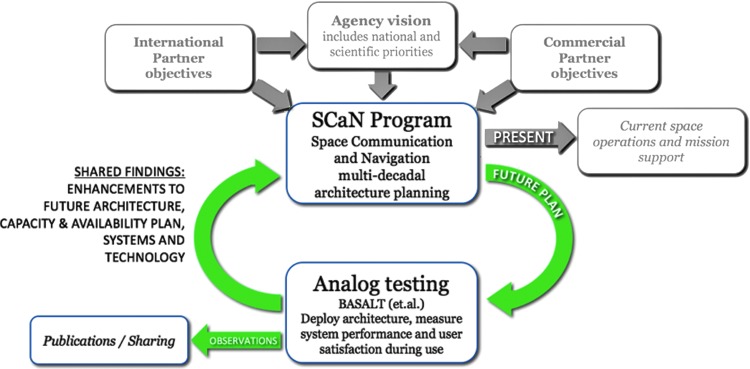
A synergistic relationship between analogs such as BASALT and NASA's SCaN Office. SCaN, Space Communications and Navigation.

In the fall of 2008, the D-RATS rovers simulated a lunar surface mission. Two prototype pressurized lunar surface rovers were roving the exploration theater and carrying two crewmembers and a large suite of support equipment generating telemetry. Each rover was sourcing and downlinking to Earth 5 Mbps of telemetry data to a nearby relay, which then aggregated those streams into one ∼10 Mbps stream to “Earth” (the control center in Houston). At that time, the only proximity radio link technology available to missions in deep space was the first versions of the Electra radio system (Edwards, 2003)—which was and is today compliant with the Consultative Committee for Space Data Standards (CCSDS)^**^ Proximity-1 (aka “Prox-1”) and is *the* standard for short-range wireless links at Mars today (surface to surface, surface to orbiter) (Consultative Committee for Space Data Standards [CCSDS], 2013). The problem was that, Prox-1 was only capable of transferring data in the range of 1–2 Mbps, and today peaks at 6 Mbps. While achieving these rates in the harsh Mars environment is a major achievement for humanity, SCaN personnel recognized that a new higher data rate proximity wireless standard was needed for future human missions. We believe that this experience solidified the beginning of a new NASA SCaN/analog relationship, which continues to this day. Figure 3 best illustrates this relationship.

## 5. Understanding NASA's Space Communication Plan for Human Exploration

A basic understanding of NASA's space communication architecture development plan is important to show the synergies present between modern analogs and program planning. NASA's SCaN Program office has an accelerated multidecade buildup plan in place that continues to evolve. It is updated publicly every couple of years as new mission concepts come to life, user and mission needs change, and as new telecommunications and navigation technologies emerge and develop (Younes and Schier, 2014).

### 5.1. Goals of the next-generation infrastructure

The overarching goals of the SCaN near-Earth and deep-space infrastructure for missions have evolved further since 2014, and the latest objectives are below (Reinhart *et al.*, 2017):

(1) “Shrink” the Solar system by connecting the principal investigator more closely to the instrument, the mission controller to the spacecraft, and the astronaut to the public.
(2) Improve the user's experience and reduce user burden, that is, reduce the effort and cost required to design and operate spacecraft to receive services from the SCaN Network.
(3) Reduce network burden, that is, reduce the effort and cost required to design, operate, and sustain the SCaN Network as it provides services to user missions with a collateral benefit of increasing related technology funding.
(4) Apply new and enhanced capabilities of commercial telecommunications and navigation to space, leveraging other organizations' investments.
(5) Enable growth of the domestic commercial space market to provide—and NASA to use—commercial services currently dominated by government capabilities.
(6) Enable greater international collaboration by establishing an open architecture with interoperable services that foster commercial competition and can be adopted by international agencies as well as NASA.

A major step was taken in the development of interoperable deep-SCaN capabilities recently. NASA's SCaN office worked with the International Space Station (ISS) partner agencies from 2016 to 2018 to jointly develop a plan to enable at least a minimum level of standardization for interoperability. In essence, if a spacecraft is built by any organization using these standards, they will be directly compatible (all interfaces, docking/physical, communications/radiofrequency [RF], navigation/tracking, etc.) with the next phase of exploration elements under development for cislunar space and beyond (Orion, Gateway, and others). All of the new standards, including the communications and navigation standard, can be found at (International Space Station [ISS] Partner Agencies, 2018).

Telecommunication links between Earth and deep-space mission elements are not normally made possible with a single radio link. Multiple independent link “segments” are combined to form a complete path between Earth systems and deep-space systems, such as complex terrestrial/fiber segments on Earth, deep-space long-range link segments between Earth and relays orbiting a planet, midrange link segments between the orbiting relay and elements on the surface, and sometimes short-range link segments between surface elements. To achieve all of the above goals, architecturally, SCaN is working multiple parallel developments to advance each major link segment or type, and so, in combination will meet user needs in the future.

### 5.2. Current and future planetary relays

The first example of this is a new approach to planetary relays. SCaN is studying Next-Generation Relay concepts (National Aeronautics and Space Administration, 2015) as (1) a replacement to NASA's current Earth-orbiting TDRS and (2) a way to standardize the relay services needed at every planet NASA explores long term, using a design that is as planet agnostic as possible, to reduce costs.

**FIG. 4.**
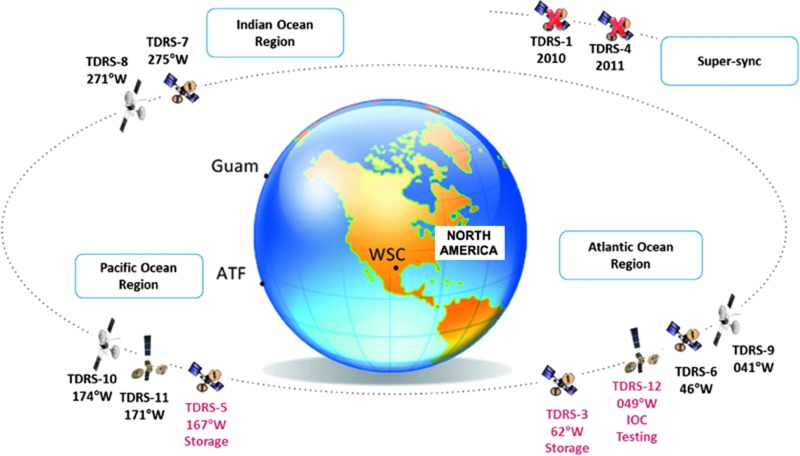
NASA's current TDRS constellation. Note: Satellite locations are approximate and vary from year to year. Updated from National Aeronautics and Space Administration (2014) image. TDRS, Tracking and Data Relay Satellite.

NASA's current TDRS relay satellite infrastructure is an array of relays deployed along Earth's geostationary ring, as shown in Fig. 4. Each satellite hugs its orbital position closely but is deliberately moved on occasion in support of NASA mission needs. As spacecrafts orbit Earth below the constellation, they are “handed off” from one active TDRS in each region to one in the next region—much like modern cellular telephones are handed off from tower to tower on the ground.

NASA's first TDRS spacecraft launched in 1983. The constellation provided near-continuous communications and tracking coverage for all the space shuttle missions. It served, and continues to serve today, the ISS, and has also supported many other spacecraft from many nations and US Government agencies in the lifetime of the system.

The TDRS constellation is capable of supporting higher data rates (National Aeronautics and Space Administration, 2001) but today provides the ISS aggregate return/data downlink rates up to 300 Mbps and up to 25 Mbps of forward/uplink rate (Roberts, 2016) data, thanks to recent upgrades on both the ISS onboard and in the supporting ground systems (Cecil *et al.*, 2012).

**FIG. 5.**
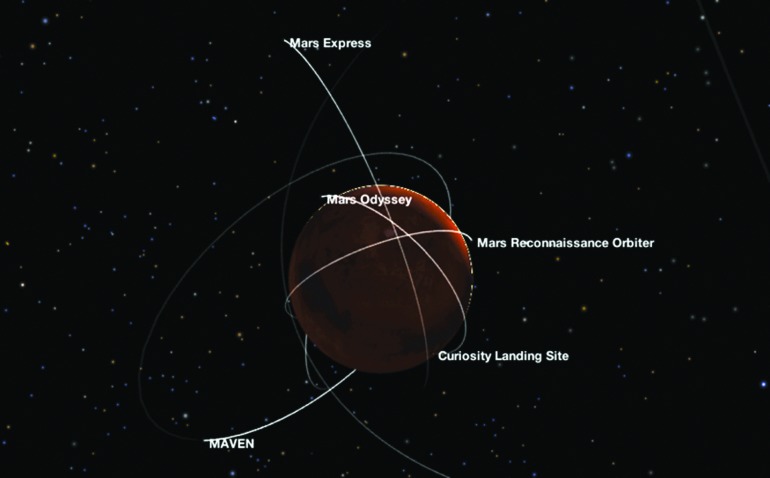
The Mars Network: Combined NASA-ESA current Mars relay infrastructure. Screen capture taken “live” in February 2018 from NASA's web-based “Eyes on the Solar System” exploration tool, online: https://eyes.nasa.gov

The Mars Network of relays evolved gradually over time. NASA's Mars Program originated the relay infrastructure to support the Mars Exploration Rovers. Science orbiters were augmented with a relay capability for others to use decades into the future (Edwards *et al.*, 2006). Today, to save infrastructure cost, all orbiters going to Mars are designed to evolve into a multidecadal telecommunication relay once the scientific experiment concludes. The current Mars Network of supporting orbiting relays (both NASA and the European Space Agency's Mars Express spacecraft) is the most capable infrastructure ever present at a planet beyond Earth and a snapshot of these assets and their diverse orbits can be seen in Fig. 5.

It is important to note that, while the Mars Network is an incredible achievement in its current form for Mars science and all of humanity (humans have a multinode network supporting Mars!), it is not without many performance limitations. Limitations on the current Mars Network make it unsuitable to support anything except very short human missions to Mars (lasting less than a few weeks). There are several reasons for this. First, as discussed earlier, the relays are primarily in fast-moving low-Mars orbits—this causes short link availability (Vuong and Vuong, 1997) that is incompatible with long-term human mission needs. Second, today when surface elements do see a relay, their maximum link rates to push data to the relay are no more than 6 Mbps—the maximum aggregate/return link data rate from Mars relay to Earth today. The third and likely most problematic performance issue with the relay capabilities at Mars is the forward/uplink capability, which falls three to four orders of magnitude short of an ability to support long-duration human exploration. The forward aggregate link to Mars is limited to <256 kbps, or 0.256 Mbps today (to all Mars assets combined at a given time). Return rates from Mars are from the 100s of kbps to as much as 6 Mbps. While data rates to Earth can be 1–5 Mbps (20–200 Gb/sol), only about 0.5 Gb/sol of that can be returned, per landed asset, by such limited relay opportunities (Chamberlain *et al.*, 2015; Lock *et al.*, 2016).

Recall that the ISS today receives up to 25 Mbps on the forward/uplink and can return/downlink up to 300 Mbps, by comparison (Cecil *et al.*, 2012). Human presence in space increases forward link demand for a variety of reasons that are beyond the scope of this article.

**FIG. 6.**
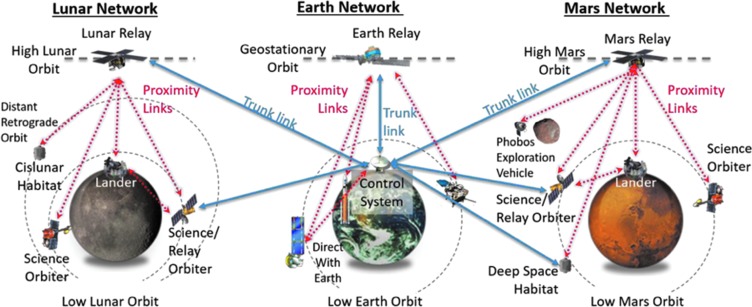
NASA/SCaN's Next Generation Relay Concept. Source: National Aeronautics and Space Administration (2015).

The Next-Generation Relay concept is an interplanetary culmination of both the mature TDRS capability and user experiences at Earth plus the also maturing relay capabilities deployed around Mars and the resulting user experiences with them (Fig. 6).

The Next-Generation Relay concept standardizes the notion of interplanetary trunk links working in concert with proximity relays parked in TDRS-like “geosynchronous” orbits but named differently: “areosynchronous” (“geosynchronous” orbits around Mars^††^). Areosynchronous orbits provide near-continuous links for surface assets on Mars with Earth (Edwards *et al.*, 2006) and are integral to future Mars surface exploration.

### 5.3. Future planetary relays: hybrid RF and optical comms

The current plan is to provide both RF and optical/Lasercomm links at the planetary relays, to enable much larger capacity trunk lines between Earth and the relays at each planet. For the relay links to ground, improvements in the Proximity-1 data link are necessary for human missions and many options are under consideration to replace the underperforming (for human missions) Proximity-1 data link option currently in use.

Lasercomm technology is proving to be very promising to enable very high data rate links to missions as far as Mars (and maybe beyond), and also being considered an option to provide aggregate trunk lines from the relays down to the planetary surfaces. As mentioned earlier, in 2013, SCaN conducted an experiment to test Lasercomm links from the Moon to Earth and back, using very small (10 cm) optics on the lunar-side flight system. The Lunar Laser Communication Terminal (LLCT) was flown on the LADEE spacecraft with the Lunar Reconnaissance Orbiter (LRO) (Boroson *et al.*, 2014; Cornwell, 2015). Optical forward/uplink rates to LLCT from Earth were up to 25 Mbps, and despite the very low-power optical transmitter on the LLCT, return/downlink data rates were 622 Mbps. NASA is investigating the potential of using 12 m optical apertures in ground stations on Earth, doing so would enable significant forward and return link rates, especially for Mars, if implemented.

### 5.4. NASA's future Mars telecommunication architecture

**FIG. 7.**
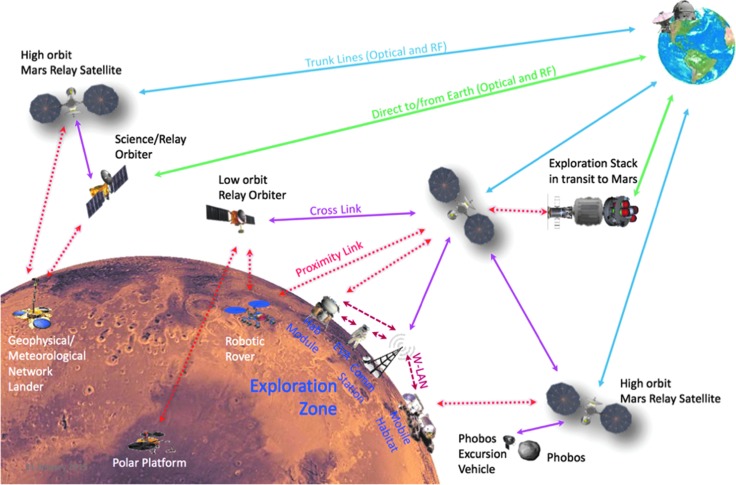
NASA/SCaN's Next Generation Mars telecommunication architecture to enable long-term human exploration. Source: Reinhart *et al.* (2017).

Figure 7 shows the currently planned, but constantly evolving, Mars telecommunication and tracking/navigation major elements. In addition to capabilities already mentioned, it is important to note that the high Mars orbit/areosynchronous relay at Mars will not only provide relay services for Mars surface assets in view but will also be able to provide relay services for missions to Phobos and crosslink service to other lower altitude relays and spacecrafts orbiting below them. Support to missions to the Mars moons would see a 50–75% availability/coverage schedule (compared with near 100% availability to surface assets in view), due to the spin of the Mars moons and assumed synchronous spin of elements exploring them.

Mars may also 1 day be enveloped in a web-like constellation of small relay satellites, also known as “smallsats.” Examples of these smallsat constellations include active commercial ventures such as “StarLink,” “OneWeb,” and others planning large-scale deployments around Earth (Shihora, 2018). These constellations of smallsats have the potential to enable truly global broadband access on Earth. If deployed in a Mars environment, they could also enable surface telecommunication coverage encompassing the entire planetary surface. They could also provide a GPS-like capability for Mars navigation, with faster position determination and higher accuracy than a few areosynchronous satellites around Mars can ever provide. It does not appear, however, that these smallsats would be equipped with the higher power direct with Earth trunk link systems, nor may it make sense for them to have this capability for power reasons alone. We believe that high-power, high-orbit relays will still play a key infrastructure role if smallsats are present 1 day at Mars. The low-orbit, low-power smallsats will benefit from the high-orbit, high-power/areosynchronous relays to aggregate their data flows to/from Earth. All these will be transparent to the end users both on the surface and on the Earth side, as the data move from relay to relay along the way.

Validation and operation of these capabilities at the Moon is, in theory, less complex than validation over long telecommunication latencies with Mars. They will also yield much higher data rates (forward and return) with assets at the Moon, and better Earth tracking than the more strenuous links to/from Mars-situated assets.

### 5.5. Risks to implementing the SCaN vision

NASA budgets as well as international and commercial partner investments in deploying future Moon and Mars telecommunication, network, and navigation infrastructure over the coming decade is critical. The success of these joint developments will (for better or worse) define how robust or constraining the Moon and Mars networks end up for human missions and their supporting teams on Earth. In addition, relay capabilities must be manifest as secondary payloads on other primary spacecrafts headed to Mars. These high-orbit relays at Mars are essential to answer both the high data rate and availability needs of human crews. Installing these relays will be highly dependent on them “finding a ride” to Mars (*i.e*., being able to be comanifest or be a direct part of other satellites launched to Mars). The cost to launch satellites to Mars is currently too prohibitive (today) for telecommunication assets alone to take a solo ride to Mars. Each mission to Mars today must have science objectives at its forefront, and if the assets can be carried along, telecommunication enhancements get a ride along too.

SCaN believes that future cargo missions to Mars carrying equipment and landers, missions that will predate crewed missions, will be a key opportunity to carry the higher power trunk link equipment and high orbit relays. These cargo spacecrafts, such as a future version of the Power and Propulsion Element (PPE)—the first element of NASA's Lunar Orbital Platform-Gateway (aka, “Gateway”) system, may solve this problem. The PPE is referred to as NASA's new “space tug” in the NASA budget (slide 3 at National Aeronautics and Space Administration, 2018b). These vessels could be modified to carry the trunk link equipment as a secondary payload, then, after completing their primary drop at Mars, loft themselves up to a high Mars (potentially even areosynchronous) orbit and park to serve as the propulsive host to the high-orbit relays. These cargo tugs will have a significant power capacity available for telecoms onboard, since these elements require significant power to drive their hybrid electrical/ion drive motors for the cruise phase to Mars.

### 5.6. Prospects for public/private partnerships to support future space communications and navigation

NASA's SCaN office released a draft of a new concept to provide SCaN services for missions and users—partnerships envisioned between NASA and one or more private sector partners. The draft solicitation, released in a NASA Broad Agency Announcement (BAA), September 18, 2018 (National Aeronautics and Space Administration, 2018c), describes NASA's vision for these public/private partnerships (PPPs) to support human and robotic missions between Earth and the Moon, out to missions as far as 2 million km from Earth. If successful, these new PPPs could create a new business model for affordable comm and nav services to missions.

## 6. Key Insights the BASALT Program and Other Analogs Have Provided to SCaN Planners

The SCaN program enhanced its architecture plan as a result of decades of robotic exploration, human lunar exploration, and new insights gained from analog field test operations since 2009, and the most recent insights have come from BASALT. The first enhancements made to this architecture plan can be seen in the most recently released Architecture Definition Document (ADD) described earlier in Younes and Schier (2014), but the impact of more insights can be seen in the more recent SCaN publications mentioned earlier (Reinhart *et al.*, 2017).

Today, SCaN is socializing an evolved version of the 2014 Architecture with some notable enhancements in the following areas:

(A) Planning for significant data movement from crews in deep space to Earth (the “return” link). BASALT has emphasized the criticality, utility, and a “discovery-multiplying”^‡‡^ effect when support personnel on Earth have access to high-capacity data downlinks during scientific EVA operations (Miller *et al.*, 2019). The need for high-capacity downlinks also became apparent in other science communities, and this need has contributed to the gradual development of a new link standard, the Unified Space Link Protocol (USLP) (Kazz *et al.*, 2016). The demand for these high-rate user downlinks was also one of the supporting needs behind the development of extremely high data rate deep-space Lasercomm technology. As mentioned above, SCaN's Optical Communication Program demonstrated very high data rates (forward and return link) between Earth and the Moon in 2013 (Boroson *et al.*, 2014; Cornwell, 2015). BASALT and many previous analogs multiplied requirements emerging from NASA's robotic science missions and helped gain support for this increased downlink data rate need.
(B) Planning for significant data movement from Earth to crews in deep space (the “forward” link). Today, robotic explorers require very little data on the forward link, normally limited to sending commands and software updates to the systems onboard (Edwards *et al.*, 2006; Makovsky *et al.*, 2009). ISS crews and analog tests revealed the need for data from Earth when humans are the explorers^§§^ (Seibert *et al.*, 2017a, 2017b). This insight has led to much greater budgetary emphasis on boosting all systems in the DSN and in-space systems that will enable large forward link capability to support human explorers. D-RATS first revealed this during overnight science team analysis activities where large amounts of mapping and other data had to be uploaded to the crew, and NASA's HRP has exposed a significant need for forward link data within its HI-SEAS project studying crews in isolation for periods longer than 4 months. Measurement of network traffic to the HI-SEAS habitat during mission 3 (Seibert *et al.*, 2017a) showed high forward link utilization for cached Internet content delivery, file sharing from ground support teams, both entertainment and work, and media and family interaction video and imagery products, and many more data types. Of special significance, HI-SEAS revealed that crews living and working on a planet surface will have the same need for access to a variety of Internet reference information to perform their jobs on a daily basis. Examples include literature searches, software code snippet searches, training and instructional information for tasks not planned before the mission, to name just a few. This likely means crews will need living/updated portions of hundreds of Internet domains constantly “trickled” to Mars, so a local copy is stored on a Mars server on the planet surface. This will enable day to day work that requires Internet reference content to be accomplished by browsing locally the most current information from Earth available. This flow of Internet content “mirroring” to crews living on a planet surface greatly increases the need for a strong forward link to the planets to keep the Mars “copy” up to date as it changes on Earth.
(C) Planning for significant improvements in link availability to destinations where human explorers are present. Link availability was introduced in Section 5.2. It describes the amount of time within a standard block of time that explorers can actually “connect” Earth to flow data each hour, or sol (martian day), or week, or month (whatever the desired figure of merit is for the mission objectives). Today's Mars surface rovers operate just fine with 8–10 minutes per sol of link availability, for example. In this article, we refer to “end to end” link availability. End to end availability is more challenging, because these link paths between deep space and Earth consist of multiple combined link “segments,” all of which must be joined and maintained continuously^***^ during near real-time, availability-demanding operations. This enables end to end connectivity so that “real-time” EVA data can flow across the segments, both ways.

## 7. Conclusion and Findings

### 7.1. Critical: Crew interaction with Earth-based teams

Before analog testing, two different belief systems seemed to emerge, that is, those who felt scientists on Earth could interact with crews, and those who felt it was not worth trying and it could all be automated.

In 1998, the Lunar and Planetary Institute, sponsored by NASA, held a science workshop about the first human missions to Mars, entitled the “Mars Field Geology, Biology, and Paleontology Workshop.” It is clear from their report that they envisioned exactly what the BASALT program is showing today experimentally, as they wrote in their summary report (Budden, 1999),

Throughout the mission, rotating teams of research specialists on Earth will be on call and responsible for specific exploration segments and/or disciplines. They will be in regular contact and would respond immediately in the event of significant discoveries (the “Eureka!” experiences) and unanticipated events. Data transmitted to Earth will be monitored and interpreted by the Earthbound experts, who will collaborate on modifying the maturing field and analytical strategies based upon evolving interpretations. Team members on Earth will synthesize and report to the crew any relevant new publications and discoveries in their respective disciplines.

However, by the early 2000s, as new computer technology emerged, new ideas of replacing humans on Earth with intelligent computers in space also emerged. Some researchers investigated this exploration model and made the case that the lengthy communication delays to planets such as Mars would make “live-delayed” EVA communication between crews and Earth so difficult that it could not be justified (Clancey *et al.*, 2007). For many years, it seemed as if Mission Operations personnel in the agency were in agreement that crews might end up basically alone in their operations on Mars and “*call us if you need us*.”^†††^

### 7.2. Long periods of daily link availability appear to be needed

Early in the 2000s, SCaN and many other programs were assuming that brief contact periods with crews might satisfy mission needs, similar to the McMurdo station link model. This is somewhat similar to how the Mars rovers operate today, with some key distinctions. Everything the Mars rovers do is carefully choreographed on the ground and uplinked to the rovers on a daily basis, as a sequence of events, and the rovers are capable of limited autonomous work outside of their assigned sequences. Humans at McMurdo station can work some of their tasks autonomously between periods of telecommunication link availability. Today's Mars rovers also operate with significantly smaller periods of contact (availability) than McMurdo crews enjoy, as described in Section 3. In any short-duration availability scenario with humans on Mars, crews will be deprived opportunities to benefit from consistent interaction with ground-based SSTs during operations such as exploration EVAs. This is a supporting reason to establish a high link availability between Earth and Mars.

The BASALT program has provided further evidence that the scientists who met at the workshop in 1998 had the right idea. The BASALT team demonstrated that live-delayed exchange of data and spoken words between human explorers on Mars and humans on Earth is possible and can be a productive exchange from both operational and scientific perspectives (described in Kobs Nawotniak *et al.*, 2019; Stevens *et al.*, 2019).

### 7.3. Programmatic impacts of our findings

BASALT research has also demonstrated the need for upgraded telecommunication capabilities for future Mars explorers particularly during EVA. BASALT findings are important to SCaN, as they show just how critical (in particular) constant link availability between Earth and the remote explorers will be, and that intra-EVA science support, for example, can be accomplished through critical networking and time-lining capabilities (Beaton *et al.*, 2019a,b). These analog studies have direct implications on the need for a more robust deep-space capacity plan, one that could lead to more antennas or new Lasercomm ground stations to be deployed at each NASA DSN site over time, and this is believed to be a significant investment. As of the date of this article, SCaN is undergoing a multiyear deep-space capacity study. We predict that the SCaN capacity studies, when completed, will reflect these findings. It takes many years, maybe more than a decade, to build up this high-capacity and availability capability, so we feel the time is *now* to expose these needs, so they can be ready for those first boots on Mars.

## Abbreviations Used

BASALT: Biologic Analog Science Associated with Lava Terrains
COTM: Craters of the Moon
CSA: Canadian Space Agency
D-RATS: Desert Research and Technology Studies
DSN: Deep Space Network
EVA: extravehicular activity
ExPOC: Exploration Planning and Operations Center
GSFC: Goddard Space Flight Center
HI-SEAS: Hawaii Space Exploration and Analog Simulation
HMP: Haughton-Mars Project
HRP: Human Research Program
ISS: International Space Station
LLCT: Lunar Laser Communication Terminal
MDRS: Mars Desert Research Station
PPE: Power and Propulsion Element
PPPs: public/private partnerships
PSTAR: Planetary Science and Technology Through Analog Research
RF: radiofrequency
SCaN: Space Communications and Navigation
SST: science support team
TDRS: Tracking and Data Relay Satellite

## Notes

^*^ Conversations with NASA Headquarters exploration planning personnel, summer of 2017.

^†^ This work has previously not been published but was accomplished by the authors.

^‡^ The potential for future space-based cloud computing clusters (Straub, 2014) near the explorers one day could also perform this type of function and reduce the amount of time required to process the imagery even further.

^§^ The International Telecommunications Union (ITU) defines the “near Earth space” region as an Earth-centered spherical volume of space with a radius of 2 million kilometers (International Telecommunication Union Recommendation [ITU-R], 2014). “Deep space” is thus any point in space that is >2 million kilometers from Earth at any given time. For reference, the limits of the near-Earth region are more than five times the distance from the Earth to the Moon. Other international organizations (geological societies, *et al*.) have slightly different definitions.

^**^ CCSDS was founded in 1982 by the major space agencies of the world, and is a multinational forum for the development of communications and data system standards for spaceflight. Details: https://public.ccsds.org/about/default.aspx

^††^ Names derived from Greek mythology: Earth = Gaia (Geo-) and Mars = Ares (Areo-).

^‡‡^ The concept of “discovery multiplying” is explained (Beaton, 2019a,b).

^§§^ Revealed in conversations about crew and payload forward-link data utilization between the first author and Boeing engineers at Johnson Space Center from December 2017 to April 2018.

^***^ It is possible that more complex link paths may ultimately exist between Earth and the deep-space crew, as opposed to a single path of combined segments. Multiple parallel paths can be used for streaming content to move between Earth and deep space. This scenario is also nominal as long as the pieces, bundles, or packets of information arriving on each end arrive in a somewhat time-sequenced manner so they can be reassembled in proper order before presenting the information stream to the recipient(s) (*e.g.*, voice, imagery, video, time-critical data products, and other telemetry).

^†††^ Opinion of a Johnson Space Center's Mission Control Center engineer, expressed to the first author circa 2004.
